# Use of a Novel Enhanced DNA Vaccine Vector for Preclinical Virus Vaccine Investigation

**DOI:** 10.3390/vaccines7020050

**Published:** 2019-06-13

**Authors:** Rosamund Chapman, Edward P. Rybicki

**Affiliations:** 1 Institute of Infectious Disease and Molecular Medicine, Faculty of Health Sciences, University of Cape Town, Observatory, Cape Town 7925, South Africa; ros.chapman@uct.ac.za; 2 Biopharming Research Unit, Department of Molecular & Cell Biology, University of Cape Town, PB X3 Rondebosch, Cape Town 7701, South Africa; ed.rybicki@uct.ac.za

**Keywords:** DNA vaccine, HIV-1, enhancer element, circovirus, immunogenicity

## Abstract

DNA vaccines are stable, safe, and cost effective to produce and relatively quick and easy to manufacture. However, to date, DNA vaccines have shown relatively poor immunogenicity in humans despite promising preclinical results. Consequently, a number of different approaches have been investigated to improve the immunogenicity of DNA vaccines. These include the use of improved delivery methods, adjuvants, stronger promoters and enhancer elements to increase antigen expression, and codon optimization of the gene of interest. This review describes the creation and use of a DNA vaccine vector containing a porcine circovirus (PCV-1) enhancer element that significantly increases recombinant antigen expression and immunogenicity and allows for dose sparing. A 172 bp region containing the PCV-1 capsid protein promoter (Pcap) and a smaller element (PC; 70 bp) within this were found to be equally effective. DNA vaccines containing the Pcap region expressing various HIV-1 antigens were found to be highly immunogenic in mice, rabbits, and macaques at 4–10-fold lower doses than normally used and to be highly effective in heterologous prime-boost regimens. By lowering the amount of DNA used for immunization, safety concerns over injecting large amounts of DNA into humans can be overcome.

## 1. Introduction

DNA vaccines were hailed as long ago as the 1990s as the next best thing in vaccines: Plasmid-based DNA vaccines are relatively easy and affordable to produce, sharing a common production method for all vaccines; they are thermostable and safe with no risk of virulence or apparently of anti-vector immunity, can be administered to immunocompromised individuals, and multiple plasmids can be mixed and used as a broad spectrum combination vaccine. DNA vaccines elicit mainly cell-mediated immune responses due to presentation of expressed antigens via major histocompatibility complex class I (MHC-I) presentation, which is similar to viral pathogens and a desirable feature of a vaccine [1]. One important drawback to DNA vaccines, however, is their lack of immunogenicity compared to protein-based or whole virus vaccines: Humoral responses are generally weak if not lacking altogether, and high, repeated doses of DNA are needed in order to obtain reasonable response rates in animal models. Additionally, results in small experimental animals have not translated well into human clinical trial results, and there are concerns over the safety of injecting large amounts of DNA (milligrams) into humans [2]. 

## 2. A Novel Enhancer Sequence for DNA Vaccine Antigen Expression

Our group therefore previously investigated the potential of short enhancer sequences derived from a mammalian single-stranded DNA virus—porcine circovirus type I (PCV-1)—for dose-sparing potential and immunogenicity enhancement in a clinically trialed HIV-1 subtype C DNA vaccine [3]. The plasmid vector (pTH) has been well used in preclinical and clinical studies [4,5,6] and is regarded as being a high-potency vaccine antigen vector for HIV and other agents. It relies on the human cytomegalovirus immediate/early promoter (CMV I/E) enhancer element constituting the promoter Pcmv [7], one of the strongest known promoters in mammalian expression systems, driving in vivo antigen expression with the help of the CMV intron A and the bovine growth hormone polyadenylation signal. It has been used to vector the synthetic HIV-1 subtype C vaccine antigen GrttnC, a polyprotein incorporating Gag, reverse transcriptase (RT), Tat, and Nef sequences, in studies in mice, guinea pigs, monkeys, and humans [8,9,10,11,12]. 

**Figure 1:**
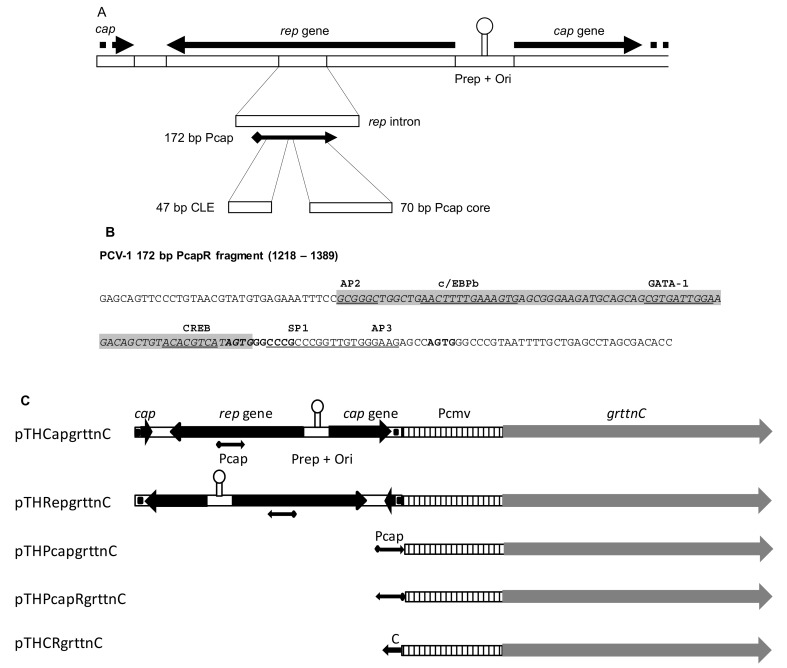
Porcine circovirus type-1 (PCV-1) genome arrangement. (**A**) Diagram of the linearized PCV-1 genome, depicted in the orientation cloned into pTHCapgrttnC. The *rep* intron is enlarged and the capsid gene promoter (Pcap) indicated. The core and conserved late elements (CLE) components of Pcap are shown. *rep* = replication associated protein gene, *cap* = capsid protein gene, Prep = *rep* gene promoter, Ori = origin of replication, core = composite host transcription factor binding site. (**B**) DNA sequence of 172 bp PcapR fragment. Putative host transcription factor binding sites are indicated and underlined, CLE motifs are in bold and the minimal PcapR sequence (1252–1238; as identified by Mankertz and Hillenbrand [13]) is highlighted in gray. PCV-1 accession number Y09921. (**C**) Schematic diagrams of plasmids showing assembly of PCV elements. Pcmv = Cytomegalovirus (CMV) promoter, *grttnC* = gene encoding polyprotein of HIV-1 Gag, reverse transcriptase (RT), Tat and Nef, C = 70 bp Pcap core. Figure reproduced from Tanzer et al. [3] under the Creative Commons Attribution (CC-BY) license as specified by BioMed Central.

**Figure 2:**
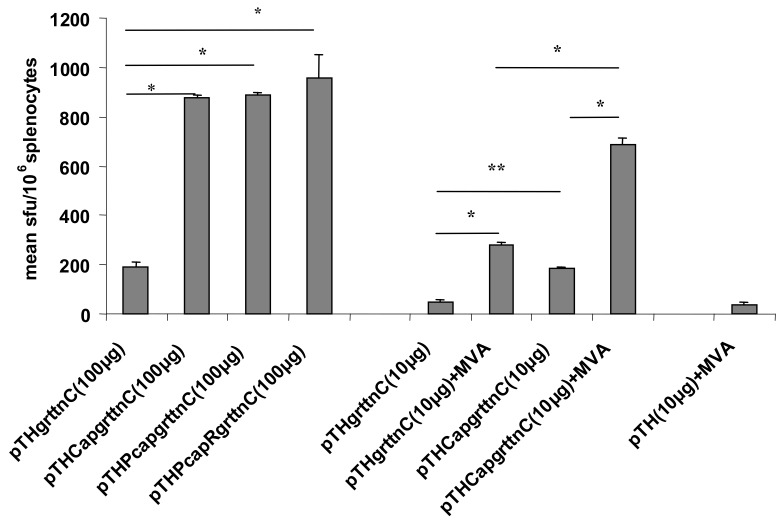
HIV-1 specific IFN-γ ELISPOT responses to pTHgrttnC DNA vaccines containing portions of the PCV-1 genome. Groups of mice were vaccinated intramuscularly with DNA vaccines on days 0 and 28. Two groups of mice were subsequently boosted with 10^4^ pfu of modified vaccinia Ankara (MVA) on day 56. A separate group of mice was vaccinated with 10 μg pTH (empty vector) on days 0 and 28 and subsequently boosted with 10^4^ pfu of MVA on day 56. * *p* < 0.001; ** *p* < 0.05 Student *t*-test. Figure reproduced from Tanzer et al. [3] under the Creative Commons Attribution (CC-BY) license as specified by BioMed Central.

PCV-1, like all circoviruses, has a compact, genetically dense, bi-directionally transcribed genome of 1759 bp that encodes only a viral capsid protein (*cap* gene) and the replication-associated proteins Rep and Rep′, which derive by alternative splicing from one open reading frame (ORF) (*rep*) (Figure 1). Bidirectional transcription of the two genes originates in the origin of replication (Ori) for *rep*, and in an intron within *rep* for *cap* [13]. In vitro expression studies in human embryonic kidney 293 (HEK293) cells with various constructs derived from the PCV-1 genomes showed that enhancement activity resided in a 70 base pair “core sequence” (C) of the 172 base pair (bp) capsid promoter, Pcap, that includes a putative composite transcription factor binding site comprising CCAAT/enhancer-binding protein beta (C/EBPb), GATA-1, and cAMP response element-binding protein (CREB) sites, as well as a 47 bp conserved late element, or CLE. Inclusion of the 70 bp sequence in the reverse orientation immediately upstream of the Pcmv sequence in pTHgrttnC (yielding pTHCRgrttnC) resulted in 2.4-fold enhancement of polyprotein expression level in vitro following transfection of HEK293 cells, as assessed by Gag p24 ELISA. The cognate sequence from the related PCV-2 was equally effective. The 172 bp Pcap sequence also enhanced luciferase expression in HEK293 cells three-fold when inserted in reverse orientation upstream of the simian virus 40 (SV40) promoter in the commercial pGL vector [3]. Accordingly, we tested the enhancement of immunogenicity in vivo by intramuscular injection of mice with a variety of pTHgrttnC constructs with additives from PCV-1 (Figure 1C): The best enhancement over pTHgrttnC, as assayed by interferon-gamma enzyme-linked immune absorbent spot (IFN-γ ELISPOT) responses to a RT CD8^+^ peptide, was obtained using the Pcap (172 bp) insert, after two intramuscular inoculations of 100 μg of pTHPcapRgrttnC DNA (five-fold increase in spot forming units (sfu)/10^6^ splenocytes). Moreover, two inoculations of 10 μg of pTHPcapgrttnC DNA was significantly more immunogenic (3.5-fold) than pTHgrttnC and boosting with 10^4^ plaque forming units (pfu) of modified vaccinia Ankara (MVA) vectoring Grttn showed the same trend (Figure 2). The response to the 10 μg of pTHPcapgrttnC DNA alone was also equivalent to or higher than to 100 μg of pTHgrttnC, indicating that significant dose sparing (10-fold) was possible for the same priming effect for a vaccine-relevant antigen. This proof that a simple enhancement could dramatically improve the functionality of a DNA vaccine vector led to its being employed in subsequent studies in our HIV vaccine research program. 

## 3. Testing the Enhanced DNA Vector with HIV-1 Subtype C pr55Gag

Strong polyfunctional CD8^+^ T cell responses to HIV-1 Gag or Gag-derived antigens have been found to be important for controlling viremia in HIV+ people who are termed “elite controllers.” Accordingly, Gag should be and often is included in candidate HIV vaccination regimes, so as to allow early clearance of infected cells at the initial sites of infection, as well as control of spread from these sites and later control of viremia [14]. A subtype C mosaic Gag sequence was chosen to increase the coverage of both CD8^+^ and CD4^+^ T cell epitopes from that of natural sequences with the hope of reducing the HIV-1 escape pathways [15,16,17,18]. Subtype C (HIV-1C) was chosen as it is the most prevalent subtype in the world, accounting for over 50% of all global infections and is the dominant subtype in southern Africa. In a study carried out by our group, the pTHPcapR plasmid backbone [3] was used to construct a DNA vaccine containing an HIV-1 subtype C mosaic *gag* gene, DNA-Gag^M^ [19,20]. 

**Figure 3:**
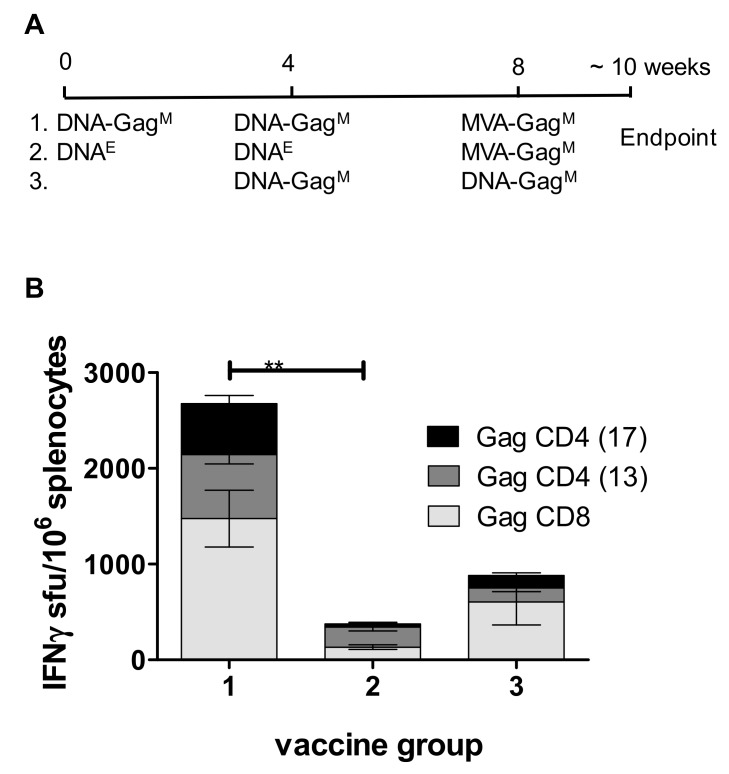
DNA vaccine elicits high Gag-specific IFN-γ ELISPOT responses both alone and in a heterologous prime boost with MVA. (**A**) Vaccination schedule. DNA-Gag^M^ = pTHPcapR containing mosaic *gag*; DNA^E^ = pTHPcapR empty vector; MVA-Gag^M^ = MVA containing mosaic *gag*. (**B**) Cumulative IFN-γ ELISPOT CD8^+^ and CD4^+^ responses of vaccinated mice to HIV-1 Gag peptides. ** *p* < 0.01 Student *t*-test of unpaired data.

HEK293T cells transfected with DNA-Gag^M^ expressed high levels of Gag (up to 26 ng/mL in the media). The immune responses to the DNA vaccine were evaluated in mice using homologous and heterologous prime boosts with MVA vaccine expressing the matching HIV-1 subtype C mosaic Gag antigen (MVA-Gag^M^). To confirm that the DNA vaccine was immunogenic at a low dose, mice were vaccinated with 10 μg of the DNA vaccine. Mice vaccinated with two doses of DNA-Gag^M^ had mean cumulative Gag-specific IFN-γ ELISPOT responses of 882 sfu/10^6^ splenocytes (Figure 3). These responses were higher for CD8^+^ rather than for CD4^+^ Gag peptides (604 and 278 sfu/10^6^, respectively). Mice that received a heterologous prime boost consisting of two doses of DNA-Gag^M^ followed by a single dose of MVA-Gag^M^ had mean cumulative Gag-specific IFN-γ ELISPOT responses of 2675 sfu/10^6^, that were evenly balanced for both Gag CD4^+^ and CD8^+^ peptides. Both the homologous and heterologous vaccination regimen elicited a higher proportion of CD8^+^ T cells expressing cytokines than CD4^+^ T cells. All the cytokine-positive CD8^+^ T cells had an effector–memory phenotype. This study confirmed that the pTHPcapR DNA vector backbone containing the porcine circovirus enhancer elicits high-magnitude, Gag-specific T cell responses in BALB/c mice at a low dose. 

## 4. Testing the Enhanced DNA Vector with HIV-1 Subtype C Env Immunogens

The kinds of immune responses that an effective HIV-1 vaccine would need to elicit include non-neutralizing antibody responses as well as broadly neutralizing antibody responses, together with polyfunctional cytotoxic T cell responses to a variety of epitopes from the HIV-1 proteome [21,22,23]. One of the main targets of recent HIV-1 vaccine candidates is broadly neutralizing antibody (bNAb) responses: bNAbs that can neutralize diverse primary HIV-1 subtype isolates protect against viral challenge in nonhuman primates (NHP) with Env-pseudotyped simian–human immunodeficiency viruses (SHIVs), suggesting that infection in humans could be similarly prevented [24,25]. Ranking of HIV-1 isolates according to their sensitivities to neutralizing antibodies allows identification of viruses as Tier 1 (sensitive), Tier 2 (moderately resistant), and Tier 3 (resistant) [26]. The circulating viruses that vaccines will need to protect against are largely Tier 2 type: Accordingly, HIV vaccines should elicit responses that neutralize laboratory Tier 2 virus isolates. We showed previously that using a DNA prime/MVA boost immunization regime in mice with vaccines expressing HIV-1 subtype C mosaic Gag resulted in strong cellular immune responses directed against Gag [19]. We wished to extend these results by improving the vaccine regimen to allow the elicitation of Env-specific neutralizing antibodies in a rabbit model. 

The pTHPcapR vector was used to construct a DNA vaccine expressing a HIV-1 envelope (DNA Env). The envelope sequence (CAP256SU) used in this study was selected as it elicited broadly neutralizing antibodies (bNAbs) in the patient [27] and was sensitive to several prototype broadly neutralizing monoclonal antibodies [28]. Several modifications were made to the envelope sequence, these included replacing the native leader sequence with the tissue plasminogen activator leader sequence, replacing the furin cleavage site with a flexible linker, introducing an I548P mutation equivalent to the I559P in the SOSIP trimers to improve the trimerization of gp41 [29] and truncating the sequence from gp160 to gp150 [30]. A second plasmid expressing the soluble envelope protein (gp140) with the same modifications was also constructed using the pTHPcapR backbone [31]. This plasmid was used to generate a stable cell line expressing high levels of the soluble HIV-1 envelope protein, which was subsequently purified and utilized as a protein boost in rabbit immunogenicity studies. MVA vaccines expressing the matching gp150 Env and Env plus mosaic Gag were also constructed. 

**Figure 4:**
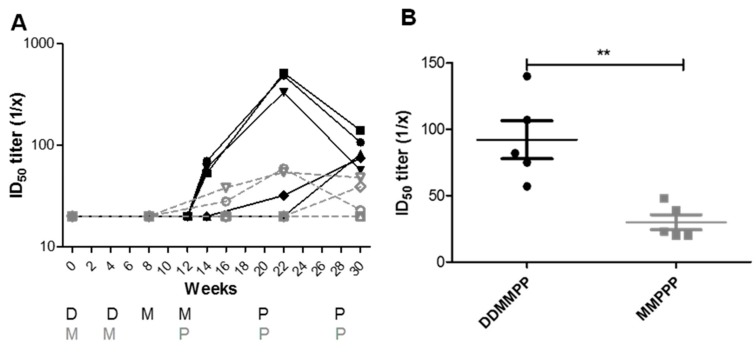
Rabbits primed with DNA produce higher autologous Tier 2 neutralizing antibodies than those receiving MVA and protein alone. (**A**) Longitudinal, Tier 2 neutralizing antibody responses to autologous CAP256SU pseudovirion from the serum of individual rabbits. (**B**) Neutralizing antibody titers at week 30. ** *p* < 0.01 Mann–Whitney U test, median of *N* = 5.

Rabbits were inoculated with different combinations of vaccines in different regimens, in order to ascertain the overall effects on immunogenicity of the Env component. The first test group was injected with 100 μg of each of DNA Env- and DNA-Gag^M^-encoding plasmids at weeks 0 and 4, boosted with doses of 10^8^ pfu of rMVA Env + Gag^M^ at weeks 8 and 12, and further boosted with gp140Env protein at weeks 20 and 28 (regime designated as DDMMPP). The other group received 10^8^ pfu of rMVA Env + Gag^M^ intramuscularly at weeks 0 and 4, followed by three protein boosts at weeks 12, 20, and 28 (MMPPP) (Figure 4).

Both the DDMMPP and MMPPP vaccination regimens elicited NAbs to the autologous Tier 2 CAP256SU pseudovirion. Moreover, high titers of antibodies that bound to the homologous CAP256 Env and a CAP256 V1V2 loop scaffold were also elicited [30]. It was noticeable that the DDMMPP regimen elicited higher mean peak titers of Tier 2 NAbs than did the MMPPP regimen: This suggests that priming with a DNA vaccine (DDMMPP) gives a better, wider anti-Env immune response than the MMPPP regime (Figure 4). The DDMMPP regimen rabbits also apparently developed a slight increase in breadth of the response as they had low levels of NAbs to clade A pseudovirus 398F1. Our findings that DNA primes a good humoral response agree with others: For example, adding DNA-C priming in the EV01 phase-I trial resulted in increased anti-Env IgG responses (from 27% for attenuated vaccinia virus strain NYVAC alone to 75% for DNA + NYVAC [32]). Priming with DNA also resulted in significantly boosted T cell responses.

## 5. Comparison of DNA Vaccines between Two Initiatives in South Africa

In 2000, a University of Cape Town (UCT)-based consortium headed by Prof. Anna-Lise Williamson was awarded funds by the South African AIDS Vaccine Initiative (SAAVI) for the development of HIV-1C vaccines for South Africa. Two vaccines—designated SAAVI DNA-C2 and SAAVI MVA-C—were deemed suitable for human clinical trials [9,10]. The vaccines expressed a HIV-1 subtype C truncated envelope protein Du151 (gp150) and the polyprotein designated Grttn described above, consisting of translational fusions of HIV-1 subtype C Gag Du422, and modified reverse transcriptase (RT), Tat-, and Nef-encoding ORFs. The vector backbone utilized for the DNA vaccines contained the regulatory R region from the 5′ long terminal repeat (LTR) of human T-cell leukemia virus type 1, which acts as a transcriptional and post-transcriptional enhancer [33]. Rhesus macaques were inoculated at weeks 0, 4, and 8 with 4 mg of SAAVI DNA-C2. No HIV-specific ELISPOT responses were detected following the DNA vaccinations (unpublished data). In a more recent study funded by the South African Medical Research Council Strategic Health Innovation Partnerships (SHIP), DNA vaccines expressing the SIV Gag and HIV-1 subtype C truncated envelope ZM109F.PB4 were constructed utilizing the pTHPcapR vector backbone (unpublished data). Rhesus macaques were inoculated, at weeks 0 and 4 with 1 mg of the DNA vaccines (four-fold lower dose). Four out of five macaques developed IFN-γ ELISPOT responses following stimulation with SIV Gag and HIV-1 subtype Env peptides. It should be noted that the antigens used in the SHIP vaccines have been designed to be more immunogenic than those used in the SAAVI vaccines and thus the improvement in the immune response cannot be solely attributed to the increased expression of the Gag and Env due to the inclusion of the porcine circovirus in the DNA vaccines. However, the SHIP DNA vaccines elicited a HIV-specific T cell response despite being administered at a four-fold lower dose than the SAAVI DNA-C2 vaccine. 

## 6. Future Possibilities for Enhanced DNA Vaccine or Expression Vectors Based on Circoviruses

Our group has recently published an investigation of the possibility of using circovirus-derived replication control elements to create replicons, or replicating dsDNA plasmid-like molecules, in plants and in mammalian cells [34]. This followed our extensive success with use of a plant ssDNA geminivirus-derived expression vector in plants as an enhanced expression vector [35]: Geminiviruses are very similar to circoviruses in having small circular ssDNA genomes that replicate via a Rep-mediated rolling circle mechanism, and very similar sequences for their non-nucleotide origins of replication (TAATATT/AC vs. TAGTATT/AC). In this study, we used a synthetic, partially dimeric clone of the genome of beak and feather disease virus (BFDV), a circovirus generically related to PCV, to investigate cross-potentiation of replication between the plant and animal viruses in plants and replication of the BFDV genome alone in HEK293TT cells.

Initial experiments where both the geminivirus-derived vector bean yellow dwarf virus (BeYDV) and BFDV genome were introduced into *Nicotiana benthamiana* plants via *Agrobacterium tumefaciens*-mediated DNA transfer showed that replication of BeYDV facilitated the co-replication of BFDV, albeit to levels only 100× less than for BeYDV replicons. More importantly, however, transfection of HEK293TT cells with the BFDV construct resulted in a ten-fold increase in genome copy number after three days. This was the first time that BFDV genomes had been shown to replicate in any animal-derived cell culture, in contrast to PCVs which readily infect a variety of cells [36]. Improvement in replicon copy number could be achieved by expressing BFDV in trans from another co-transfected vector with a strong promoter: This is not surprising, considering the native *rep* promoter is quite weak and is probably not well recognized in mammalian cells, meaning expression in trans could mean a far higher availability of Rep.

These results open up a number of fascinating possibilities for using BFDV-derived sequences as replication-competent DNA expression and vaccine vectors, several of which we are currently investigating (W. de Moor, G. Regnard, A.-L. Williamson, E.P. Rybicki, unpublished results and ongoing work). There are currently no small DNA virus-derived vectors in use in vaccinology, other than recombinant adeno-associated viruses (rAAV), and AAV2 has recently been implicated in insertional mutagenesis in human hepatocellular carcinomas [37]. Papilloma- and polyomaviruses are also known to be associated with cancers, which may preclude their use as replicating vectors. The essentially ubiquitous ssDNA torque teno viruses are potentially associated with some human disease conditions, although causation is not proven [38,39]. 

Circoviruses have never been implicated in any human disease: Although PCV-1 and PCV-2 were famously discovered in live rotavirus vaccines given to millions of children [40], and PCV-1 was shown to be able to infect a human hepatocellular carcinoma cell line [41], there was no evidence that PCV-1 infected the infants given the contaminated rotavirus vaccine [42]. There have been concerns, however, over ssDNA viruses of pigs associated with xenotransplantation in humans [43], and swine–human contacts are frequent and worldwide in agriculture. Thus, use of a circovirus such as BFDV as the source of elements for a replicating DNA expression vector, when the virus is host-restricted to one type of birds and has never been associated with human disease, is probably more likely to be regarded as safe. Our preliminary investigations have revealed considerable promise in this regard; however, these will be reported elsewhere (W. de Moor, G. Regnard, A.-L. Williamson, E.P. Rybicki, unpublished results).

## 7. Conclusions

It has been over 25 years since DNA vaccines were first introduced and many advances have been made in the field. However, despite showing promise in small animals, with some DNA vaccines being licensed for veterinary use [44,45], no DNA vaccines have been licensed for human use as immunogenicity is still relatively poor. Thus, a great deal of research has gone into improving the immunogenicity of DNA vaccines. Some of the strategies that have been shown to be effective are: (i) RNA optimization to remove mRNA structures that inhibit ribosomal loading and sequences that inhibit nuclear export of mRNA [46,47]; (ii) codon optimization [46,48,49]; (iii) use of Kozak sequences [50]; (iv) use of leader sequences to improve stability, translation, and secretion [46]; (v) use of 3′ untranslated regions (UTR) such as polyadenylation signals and post-transcriptional response elements which are important for nuclear export, translation, and mRNA stability [51]; (vi) use of different promoters and enhancers [52,53,54]; (vii) the inclusion of genes expressing immunomodulatory molecules in the plasmid vector such as GM-CSF or IL-2 [55,56]; (viii) formulation of DNA vaccines in lipids and polymers [57]; (ix) use of better delivery systems [58,59,60]; and (x) use of suitable adjuvants [58,59].

In this review, we have only focused on a single method of improving DNA vaccine immunogenicity. This was the use of a short enhancer sequence derived from the circovirus PCV-1 capsid gene promoter to increase recombinant antigen expression. This enhancer element led to increased antigen expression and immunogenicity of HIV-1 subtype C candidate DNA vaccines and allowed for the use of 10-fold lower doses. The improved performance of the DNA vaccines with these candidates, compared to non-enhanced vectors that went into human clinical trial, has prompted the inclusion of the enhancer into all DNA vaccines under investigation in our research group, with excellent results. Future use of replicating circovirus-derived DNA expression and vaccine vectors may yet open up even more exciting possibilities.
